# Research agenda for algorithmic fairness studies: Access to justice lessons for interdisciplinary research

**DOI:** 10.3389/frai.2022.882134

**Published:** 2022-12-21

**Authors:** Laura Kontiainen, Riikka Koulu, Suvi Sankari

**Affiliations:** ^1^Faculty of Law, Helsinki Institute for Social Sciences and Humanities, University of Helsinki, Helsinki, Finland; ^2^Faculty of Law, University of Helsinki Legal Tech Lab, Helsinki, Finland; ^3^Faculty of Social Sciences, Institute of Criminology and Legal Policy, University of Helsinki, Helsinki, Finland; ^4^European Law, University of Helsinki Legal Tech Lab, Helsinki, Finland

**Keywords:** law, algorithms, Artificial Intelligence, technology governance, access to justice, interdisciplinary research, method, decision making

## Abstract

Access to justice is one of the fundamental legitimating principles underlying all modern Western legal systems, yet its role in critical algorithm studies remains underdeveloped. In historical and methodological terms, the access to justice movement showcased multi- and interdisciplinary research on legal phenomena. We argue that interdisciplinary research on AI ethics and regulation, datafication of society, and algorithmic governance could benefit from adopting access to justice as a vantage point for bridging the different approaches in the context of administering justice. To this end, we explore technological, legal, and societal intersections to demonstrate how law, social sciences, and algorithm studies could benefit from a historically more informed and holistic approach facilitating more “cost-effective” interdisciplinary research collaboration. Such approach could assist the substantive study of algorithmic fairness to contribute actionable systemic solutions on what we perceive as systemic challenges. We propose utilizing access to justice as a boundary object for interdisciplinary dialogue over algorithmic fairness while respecting the epistemic diversity of disciplines.

## 1. Introduction

Algorithmic systems are increasingly impacting our everyday lives, leading to growing concern about the protection of fundamental rights (see e.g., EU proposal for a Regulation on Artificial Intelligence [COM (2021) 206, “AI Act”]. Worrying examples of algorithmic discrimination (see e.g., Burkell, 2019) have led first to the rise of AI ethics research and policy and then to a critique of industry-driven “ethics-washing” in general and the narrow scope of ethics guidelines in particular (Bietti, 2020; Hagendorff, 2020). Similar concerns for unfair treatment also feature strongly in policy-making, where they are used to justify regulatory interventions.

Considering algorithmisation overall – as a phenomenon – and regulating it, the ends and means seem incommensurable. Algorithmisation is creating systemic problems, whereas the (legal) solutions offered are piecemeal at best. Problematising issues in algorithm studies as well as novel regulation are both focusing on specific technologies or technical issues, instead of developing an understanding of how existing legal orders already regulate algorithmisation (Viljanen and Parviainen, 2022) or how they should do it. However, viewed as structural intertwined institutional- and individual-level issues, are the problems related to algorithmisation really new? Where, by whom, and how should they best be resolved?

We argue these questions should be addressed by research. Therefore, in this article we lay out a research agenda on algorithmisation and especially algorithmic fairness. We suggest that one way fairness has in the past been fruitfully deployed in the context of administering justice (i.e., making legal decisions) is in “access to justice” studies. We approach algorithmisation and algorithmic fairness as a wicked complex societal problem (Selbst et al., 2019; Woodruff, 2019), in need of interdisciplinary research. For us, algorithmic fairness is broader than the quest to reduce bias in algorithms. The concept of access to justice we use in two senses. First, it is a goal or a value and prior discussions around it provide useful common ground for contextualizing issues of algorithmisation. Second, it is a research tradition on which we draw ways of studying said issues.

In essence, this article aims to make the point that research on algorithmic fairness can be improved upon. To make this argument, we cannot separate the substantive and the methodological side of research. Nor can we neglect relevant historical perspectives. Hence we deal with all these sides below. However, this article is a prospective scoping exercise. We do not assess all possible ways to approach the phenomenon of algorithmic fairness or suggest the best one. We aim to make a convincing argument for overcoming disciplinary siloes in this field of research and offer only one practical example of many possible ways of how it could be done.

We argue that refocusing discussions on algorithmic fairness from the vantage point of access to justice could help bring together various research strands. We stress the need and method for interdisciplinary dialogue between law, social sciences, and critical data and algorithm studies. Such collaboration and discourse should offer added value in terms of scientific excellence and impact to all disciplines involved. For example, working together with legal science yields an improved and nuanced understanding of legal framings for other disciplines engaged in researching legal phenomena. Law scholars benefit from such knowledge exchange by ensuring an apt understanding of the social and technological complexity necessary to, for example, guide future law making. For scholars from other disciplines our socio-legal perspective on algorithmic fairness can seem like a mix of prescriptive and descriptive study of law and regulation. This is true, as for us, law generally retains some aspects as social engineering (Pound, 1942): the end of law is justice–in this case algorithmic fairness–which means analyses and interpretations of law inherently mix description and prescription. More specifically for our legal perspective, our objective is two-fold: both substantive, i.e., to contribute to critical research on algorithmisation; and methodological, i.e., to elaborate how to engage in the interdisciplinary dialogue needed to address algorithmisation.

We structure our argument in three steps: history; substantive context; and methodology. First, we explain how the access to justice movement (and research) originally emerged as a form of activism and scientific critique. It was directed against institutional and legal structures that render legal protection disproportionately difficult for some groups and individuals. We argue that adopting this kind of more holistic perspective–substantively and methodologically–would introduce valuable insights to support similar contextualization in the algorithmic fairness debate. This structural perspective prepares the ground for making connections between earlier access to justice research and potential for furthering algorithmic fairness. Second, from our legal perspective, we draw on the current legal discussions on algorithmisation, in the context of making–broadly speaking–legal decisions by relying on autonomous data-driven computational predictions. As substantive examples, we pick up on three topical themes discussed in the proposal for an EU AI Act: (1) transparency and explainability; (2) human agency and oversight; and (3) algorithmic fairness. We aim to demonstrate how such legal debates frame the issues of algorithmisation and hence shape its direction. Thereby, we hope our examples allow for others in social, computer and data science to pick up on similarities with approaches focusing on the same issues. Ultimately, all these themes discuss the age-old issues of fairness, already extensively elaborated within legal studies. Here, we locate the “problem” of algorithmisation in the inherent qualities of technological structures that fundamentally differ from those of legal structures. Simply put, computers are central to technological structures, whereas legal structures typically assume human agents. Third, to offer a basis for interdisciplinary work on algorithmic fairness beyond our substantive examples, we elaborate the methodological side of holistic interdisciplinary exchange. Methodologically, we propose access to justice as a boundary object and grounded theory as tools for undertaking interdisciplinary research that adds value for each participating discipline, while respecting the epistemic diversity of disciplines.

## 2. Access to justice as a vantage point

Here we introduce access to justice studies in order to draw together our argument on the several similarities between them and algorithm studies in section 3. Furthermore, we speculate that engaging with what we can learn from access to justice studies can provide significant insights into the structural challenges imposed by algorithmisation overall as well as the potential to complement both existing legal research and provide new venues for interdisciplinary collaboration.

Our suggestion of access to justice as a vantage point to algorithmisation has less to do with (re)defining a nuanced concept of access to justice and more to do with modeling the movement's successful multidisciplinary approach as well as deploying the notion as a methodologically valuable boundary object. Our notion of access to justice is one profoundly different from what the broad fundamental right of effective access to justice nowadays is in legal parlance, encompassing the duty to give reasons as part of the right to a fair trial (Council of Europe, 2008). It also comprehends the object of study more broadly than, for example, research focusing on improving access to online courts (e.g., Donoghue, 2017; Sela, 2021). In the classic (legal) access to justice literature of the 1970–80's, striving for *fairness in administering justice*, we identify a vague enough yet plausibly deployable notion of access to justice: (*1) a legal system (courts) equally accessible to all, and (2) the results of which are individually and socially just* (Cappelletti and Garth, 1978).

Applying access to justice in this sense (equal access and structural perspectives) as a boundary object could facilitate useful interdisciplinary inroads into studying *algorithmic fairness*. The notion of access to justice is more concrete than, for example, transparency or oversight (Koulu, 2021), but vague enough (i.e., a not too fixed and legally hegemonic concept) to accommodate different meanings and the epistemic pluralism necessary for interdisciplinary work. More tangibly, in methodological terms, the access to justice movement showcased multi- and interdisciplinary research on law. Therefore, we argue that applying the general notion of access to justice to focus on administering justice fairly could once again enable and form a basis for interdisciplinary collaboration in a new substantive context: It could help to further explicate the connections between law and algorithms and fairness. We suggest that such reframing of how to look at algorithmisation and algorithmic fairness would lead to more holistic studies capable of yielding more systemic solutions to present and future problems than the present siloed and fragmented approaches. Unlike some fixed and legally hegemonic concept such as fair trial or legitimacy, using the rather general concept of access to justice could facilitate the translation of existing knowledge between different scientific fields and provide a joint methodological starting point for production of new interdisciplinary knowledge.

In addition to reframing the overall approach to algorithmisation with access to justice, we also suggest reframing the relationship between technological change and law. Earlier research has contested the common misconception that law always lags behind technological progress. The STS-oriented legal scholar, Julie Cohen, refers to this as the dynamic reciprocity of law and technology, i.e., how law regulates technology development and adoption in the society but is, in turn, shaped by the same phenomena (Cohen, 2019). Technology has repeatedly affected the administration of justice and access to it before algorithmisation (Koulu, 2021) as well as through it (Donoghue, 2017). Hence algorithmisation takes place against a historical context of technology adoption and within existing social and legal structures. Both generally observing these structural underpinnings between justice and technology as well as specifically paying attention to past technological changes in the administration of justice bridges algorithmisation with insights elaborated in earlier research on access to justice.

How have access to justice studies affected law, society, and science? Originally, access to justice research emerged as a form of activism and as a scientific critique of institutional and legal structures. It built on the observation that for some groups and individuals these structures rendered legal protection disproportionately difficult. The contributions access to justice studies have made in science and society are considerable. Access to justice studies introduced agency (individual, institutional, and lawyers' perspectives) and the importance of practical and structural obstacles for exercising legal rights to complement the normative analysis of the field.

Moreover, methodologically it introduced into socio-legal research empirical knowledge about how justice is actually rendered. For example, Harold Sigall and Nancy Ostrove conducted psychology experiments with undergraduate students in laboratory settings. They observed that attractive criminal defendants would receive more lenient judgments for their crimes than others, at least in front of mock juries (Sigall and Ostrove, 1975). In similar mock trial settings, Alan Mazzella and Ronald Feingold conducted a meta-analysis on the effects of physical attractiveness, race, socio-economic status, and the gender of defendants and victims. They found that most of the time it paid off for defendants to be attractive, female and of high socio-economic status, although this also depended on the crime (Mazzella and Feingold, 1994).

In turn, Gregg Relyea examined deaf defendants' access to courts and observed how their experience of court ritual was affected by being mediated through interpreters, leading to the conclusion that the lack of language assistance rendered their procedural rights ineffective (Relyea, 1980). Through interviews with recipients of social welfare benefits, Austin Sarat was able to observe how the welfare poor encountered and experienced the dominance of legal rules differently than other groups. For them, law was immediate and powerful, ever present even in the intimate running of their lives (Sarat, 1990). To summarize, access to justice studies reformulated the barriers of access to justice more widely than just normatively, such as by a lack of resources or knowledge of the system that can prevent the individual from pursuing justice (e.g., Resnik, 2018).

As to the quintessential importance of interdisciplinary exploration of access to justice and the effects of its research results on society, Bryant G. Garth states: “Without empirical work informed by sociological theory, the access to justice movement, however much legal rhetoric goes into it, is bound to do very little for the ostensible beneficiaries of the programs.” (Garth, 2009, p. 259). As to changing the legal system, the access to justice critique that started in the 1970's by focusing on access to courts, has since the 1980's affected the way to administer justice, especially when introducing alternative dispute resolution (ADR) (Galanter, 1974; on the social psychological perspective on procedure and experience of justice, see e.g., Lind and Tyler, 1988). As to the renewal of science, the major contribution of ADR research was to challenge the hegemonic institutional perspective adopted in legal research. It shifted the object of analysis from the institutional to the perspective of the individual.

For us, the most obvious link connecting access to justice and algorithmisation studies is the recognition of structural problems, for example discrimination. To put it concretely, discrimination of certain types of individuals seeking justice is an age-old structural problem that is renewed and reinterpreted in algorithmisation. The potential of algorithmic systems to amplify existing societal biases should also be seen as a structural problem (e.g., Luusua and Ylipulli, 2020). Here we see a blatant similarity between studies of access to justice and algorithmic fairness. Moreover, legal studies alone can hardly produce the empirical, social, and computer science perspectives necessary for a further understanding of algorithmic challenges and required regulatory solutions. An obvious need for further interdisciplinary work remains.

Access to justice discourse traditionally examined phenomena on an institutional or individual level – or both from an institutional as well as an individual point of view. The algorithm studies discussion, for example on AI ethics, can make use of the same distinction. On the institutional level, unclear and difficult-to-use systems can easily lessen transparency and the individuals' trust in that they are gaining justice, whereas a well-designed system could strengthen trust. This has been noted especially in transparency discussions, where one thread has been transparency by design. Many scholars have argued that the design itself is never neutral, incorporating the values and ideologies of those involved in the design process (see e.g., Mumford, 1964; Laudon, 1974; Winner, 1985; Nissenbaum, 2005). However, on the level of individuals, the requirement of using technological tools can be a barrier for accessing justice; one must know how to use the tools and have access to them. Because of the context-dependency of ‘good design’, AI ethics research also highlights the importance of empirical research in finding out context-specific constraints and implications (Koulu, 2021).

Whether impediments to access justice remain the same or expand with algorithmisation can be revealing. Answers to this question may suggest that while the technology might be developing quickly, it is not, as a societal phenomenon, unique. Therefore, a focal question to ask is what exactly is changing with technology and algorithmisation, and what is not (see e.g., Koulu, 2020a,b). Such a historically and contextually informed enquiry into algorithmic fairness may produce more systemic knowledge on access to algorithmic justice, in turn laying the foundation for more systemic remedies to existing and novel problems.

## 3. From transparency to human oversight: Framings of algorithmic challenges and their regulatory solutions

In this section, we describe how law and technology research frames challenges and solutions related to algorithmisation in terms of transparency and explainability, human agency and oversight, and algorithmic fairness. These are issues raised also in the EU's AI Act proposal. As the perceived problems and the proposed solutions are connected, these examples showcase how recent legal research approaches problematic characteristics of algorithmisation. Although our story is told from the legal perspective, we trust that these examples offer added value for non-lawyers, as they emphasize the general thematic overlap between different fields of algorithm studies. As stated above, we do not draw a clear line neither between the substantive and methodological side of studying algorithmic fairness, nor the present and historical perspective into it, nor the descriptive and prescriptive analysis of justice/fairness. After each presentation of the *status quo* below, we suggest what the discussion could gain from adopting what we call an access to justice approach.

### 3.1. From transparency to explainability

Much socio-legal research (as opposed to doctrinal black letter law research) on law and algorithms has focused on the so-called ‘black box problem’ (also Winner, 1993; see, 1993 Pasquale, 2015). The black box problem refers to the difficulties in knowing causally how a certain output is derived from a given (data) input. Part of the challenge seems to be what exactly should be explained and to whom. Is the aim to produce causal knowledge in order to explain how a computer (algorithm) reached a given output? Or is the goal to explain how people, institutions, or the law work? Or is it to produce legal knowledge on how a decision is justified? It also matters a great deal for whom information is being produced: for the individual applicant, for an appellate court, for the deciding institution, or for an ombudsman. Generally, legal processes and their outcomes need to be understandable for laypeople as well as for legal professionals. However, focusing on the legal protection of individuals tends to overlook issues concerning power structures – a risk that access to justice studies have long since identified. In algorithm studies, these central points of access to justice studies are rarely well explicated.

Proposed solutions to the black box problem gravitate from transparency of datasets and source code to explanations on the algorithmic logic given to those affected by algorithmic decision making (Brkan, 2019; Casey et al., 2019; Käde and von Maltzan, 2019). Making algorithmic systems transparent may be more useful from an institutional point of view, but not as useful for the individual. Revealing data and explaining causality do not guarantee the understandability of algorithmic legal processes to laypeople. Increasing transparency by purely making the code visible, or even providing a summary of the logic a system works by, might not help an individual in challenging a decision made by the system. For example, without relevant knowledge of what the result of the system (the decision) would be if the system worked as it should, it is difficult for individuals to prove that they have been discriminated against, or that the system has otherwise made a wrong decision against them (Castets-Renard, 2019).

Transparency and explainability are both terms connected to potential contestation: to questioning the result, and to seeking and receiving legal redress when it is needed. These points are also relevant for access to justice and the right to a fair trial. However, algorithmic transparency and explainability differ from the traditional duty to state reasons, and their potential has been questioned. As to problems with operationalising algorithmic transparency and explainability, some legal scholars have drawn attention to the risks associated with algorithmic transparency, especially with regard to trade secrets and competition. That is, businesses might suffer from competitors gaining too open an access to their algorithms (Käde and von Maltzan, 2019). Hence, creating possible working models for explanations involves balancing what is enough information for the protection of an individual subject of an automated decision, but not too much, for the sake of businesses producing algorithmic systems.

Some have suggested that an understandable explanation does not necessarily have to be easily operationalised in practice (see e.g., Selbst and Barocas, 2018 and Coglianese and Lehr, 2019). For example, Wachter et al. have examined counterfactual explanations aimed at providing the data subject information on key variables that result in different decisions. They propose that in the face of operational difficulty, counterfactual explanations could provide a better understanding of the reasons why the decision was what it was, without actually having to make the algorithmic decision-making system transparent or explainable as such (Wachter et al., 2018). Olsen et al. have also argued that from the perspective of individuals, it does not help them to require a more detailed explanation for algorithmic decisions than is currently required from human decision makers (Olsen et al., 2021). Standards for explanations vary depending on the context. As Doshi-Velez suggests, more effort should be placed on explanations when the decision at stake would have a grave impact, would involve a large margin of error, and is hard to contest. For example, in asylum processes individuals are often exposed when compared with institutional actors in charge of the process and can find it hard to challenge or overturn a negative decision, with grave consequences (Doshi-Velez et al., 2017). As Casey and others observe, in algorithmic decision making this undue burden on the individual could at times be countered by pre-emptive measures that already justify the decisions of the system (Edwards and Veale, 2018; Casey et al., 2019).

Some practical solutions limiting the scope of what needs to be explained, such as Wachter et al.'s counterfactual explanation or who should have testing access to the algorithms, have been proposed (e.g., Ananny and Crawford, 2016; Buiten, 2019; Brkan and Bonnet, 2020). Their variety implies the rapid development and deployment of algorithmic systems across society and prolific research activity around these topics. The diversity of deployment contexts suggests little hope of a single one-size-fits-all model for balancing competing interests. However, all algorithmic explainability and transparency challenges as well as practical solutions to them relate to the overall legitimacy of decision making as well as to the individual subjects' ability to exercise their legal rights. Also institutionally, explaining the logic of the algorithmic system itself – pre-justified before any decision is made – is connected with the legitimacy and public approval of a system. Institutional legitimacy hinges on presenting a convincing logic for the system. Approval of the system on the individual level is enhanced if those subject to its decisions agree with its logic. Moreover, Selbst and Barocas conclude that the acceptability of the result of proceedings, no matter what their content, is improved by *ex ante* tying the justification of the algorithmic system logic to general principles of procedural justice (Selbst and Barocas, 2018).

In terms of individual and institutional asymmetries of power, explanation empowers individuals to act, but it also places an undue burden on the individual. This is a familiar criticism in access to justice studies (e.g., Blasi, 2009), and one worth algorithmisation studies to recognize. Moreover, drawing on access to justice studies could help algorithmisation studies to re-focus on: studying people instead of computers; actual opportunities to realize legal rights; and the overall fairness of algorithmisation.

To conclude, transparency and explainability are inherently linked with accountability and legitimacy. Transparency as such is often seen to provide justification: for example, Gaon and Stedman argue that high transparency in the government adoption of new technological innovation is necessary for the inherently valuable accountability that the government needs to have for its use (Gaon and Stedman, 2019). In contrast, Koivisto critiques such legitimation through transparency by arguing that transparency is inherently performative in nature – that institutions have an interest and need to show a carefully curated image of transparency if they wish to uphold their legitimacy in the eyes of the people (Koivisto, 2020). Overemphasis on transparency and explainability over other important principles in the use of algorithmic systems, such as accuracy and fairness, has been criticized (Selbst and Barocas, 2018). Fairness especially is a contextual concept that remains impossible to define and therefore hard to code into an algorithmic decision-making system. This is one reason why legitimacy and accountability expectations shift to human agency and oversight of algorithmic decision making.

### 3.2. Human oversight instead of explainability?

An avenue of legal research to algorithmisation deals with the role and value reserved for the human decision maker. This is a strand distinct from human replaceability (Pasquale, 2020) and from uncertainty and the risk inherent in both (AI) design as well as human decision-making and experience (Luusua and Ylipulli, 2020). The dilemma is two-pronged. First, if the presence of human agency in decision making is considered vital, lack of it would entail a total prohibition for automating certain forms of decision making. Transparency and explainability are to no avail if the value of a human being as the decision-maker – based on the human capacity for “moral questioning” (Sheppard, 2018), or “human discretion” (Davis, 2018 see also Sourdin, 2018) – is seen as intrinsic. Second, the mainstream approach to human oversight clearly builds on a strong underlying dichotomy between humans and computers. Hence, discussion focuses on algorithmic systems as entities and at most the interaction between computers and their human operators. How algorithmic systems affect human-to-human interaction remains under-researched – again an aspect much more prominent in access to justice studies by way of comparison.

Especially in legal processes, overall legitimacy and acceptability rely on the discretion of an individual judge or a panel of judges – on the intrinsic value of human beings as decision-makers. This is one reason why recent socio-legal research has questioned whether new technology can be retrofitted to the existing legal structures (e.g., Wernick and Klünker, 2019). From a legal viewpoint, the fundamental value assigned to human decision-making is visible in many debates on automation. Moreover, existing and emerging regulation advocate human oversight as a key requirement for acceptable algorithm use [e.g., GDPR article 22, COM (2021) 206 AI Act]. Whether human oversight can live up to these expectations is less clear.

When the idea is to improve the legitimacy and accountability of algorithmic systems by reintroducing the missing human back into them, the question understandably becomes how human decision makers interact with algorithmic systems. Building her argument on procedural justice, Vanderstichele argues that predictions provided by algorithmic systems, e.g., recidivism risk scores, do not seamlessly fit in with the ways in which other forms of information are interacted with and given weight within the judicial system (Vanderstichele, 2019). There are no clear answers to guide to what extent judges should rely on algorithmic predictions. Reassessing existing normative roles of different information sources in the judicial system could also work to safeguard the judge's discretion. To avoid juxtaposing algorithmic systems and human discretion, Sourdin suggests hybrid models of decision making, in which humans and algorithmic systems work together to reach the best possible outcome (Sourdin, 2018).

Both Vanderstichele's and Sourdin's approaches aptly highlight the point of understanding how human decision makers interact with algorithmic systems. Such hybrid models may seem robust enough to complement human characteristics with algorithmic systems. However, some have questioned whether the role of a human operator in charge of an algorithmic system would entail unreasonable responsibility for humans (Wagner, 2019). Despite the best intentions, the involvement of a human in the decision making can remain nominal in automated or quasi-automated systems. The phenomenon when human operators trust the system too much to exercise their discretion even when the procedural rules would leave room for it, is called “rubber-stamping.” Research has shown human reliance–possibly over-reliance–on computers and algorithms increases with the difficulty of tasks (Logg et al., 2019; Bogert et al., 2021).

As to the second prong of human oversight or ‘missing human’ dilemmas, algorithmisation discussions do not seem to pay sufficient attention to how deployment of algorithmic systems and digital interfaces affects interaction between humans. When compared to access to justice studies, this reveals a blind spot in algorithmisation studies. In access to justice, procedural justice is a key concept employed for studying the interpersonal aspects of administering justice that do not fall under outcome-oriented distributive justice (Lind and Tyler, 1988). Hence it seems the present approach in algorithmisation studies overemphasizes individualism and downplays the lived human experience of fairness and justice.

### 3.3. What about algorithmic fairness?

Finally, let us focus on fairness as one promising approach to algorithmic systems. Could striving for fairness counter the unwanted consequences of algorithms in administering justice? Could fairness be a novel point of entry, able to elevate answers to problems created or amplified by algorithmisation from fragmented juridical ones to more systemic solutions? Fairness as a holistic theme is an all-encompassing and worthy ideal to aspire toward but difficult if not impossible to implement. Fairness, as a concept, is hard to define (see e.g., Butterworth, 2018; Abu Elyounes, 2020; Nachbar, 2020), both in general and because it can be very context-specific. For example, philosophy or legal theory has little to contribute by way of an operationalisable concept. Algorithmic fairness, though a worthy ideal to pursue, is a moving target.

What seems clear based on legal literature is that algorithmic fairness is not reduced to a question concerning biased algorithms. For example, research on data processing, for example, analyses the potential of the fairness principle [GDPR Art 5(1)(a)] in interpreting legitimate interest data processing [GDPR Art 6(1)(f); e.g., Clifford and Ausloos, 2018; Wrigley, 2021]. Moreover, research suggests that the Unfair Commercial Practices Directive (UCPD) already sets a relevant standard of (un)fairness with regard to algorithmic manipulation (Hacker, 2021a). In a somewhat similar vein, if we focus on algorithmic fairness as the resurgence of a more structural fairness standard the approach gains feasibility and traction.

Consider, for example, a classic access to justice definition of fairness: a legal system (courts) that is equally accessible to all, the results of which are individually and socially just (Cappelletti and Garth, 1978). Unlike the traditional access to justice approach, AI ethics research sees fairness as more of an ideal or goal than a practical guideline. The algorithmic fairness discussion was initiated by observations of algorithmic bias (see e.g., Crawford and Calo, 2016; Ohm and Peppet, 2016; O'Neil, 2016). Understanding this general framing helps explain why law (alone) struggles to provide solutions to even this narrowly defined problem. Among others, Sandra Wachter, Brent Mittelstadt and Chris Russell have argued that the intuitive understanding of discrimination in algorithmic systems is part of why the European non-discrimination law is not equipped to deal with it. This causes difficulties for individuals who first must be able to recognize they are being put to disadvantage and then contest the treatment (also Hacker, 2018; Wachter et al., 2021; see n AI training data, Hacker, 2021b).

For us, it seems that studying algorithmic fairness as is currently done inevitably leads to attempts that divide fairness into smaller, more digestible pieces. In turn, this tendency to break fairness down into manageable bits is visible in the proliferation of lists of ethical principles for AI use. AI ethics has become popular in recent years, and the ethical guidelines and principles it has developed have become risk-management methods for the users and designers of algorithmic systems. Legal research has had fairly little to offer in terms of unpacking or defining algorithmic fairness, unlike for example bio-ethics, which has its own ethical principles built on values such as beneficence, non-maleficence, autonomy, and justice (Floridi et al., 2018). As working with the loose definition of fairness can be difficult, the same end has been sought by means of guidelines (e.g., minimizing harm, Altman et al., 2018) and legal prohibitions (e.g., anti-discrimination, Nachbar, 2020).

To us, it seems that the discussion on algorithmic fairness is still searching for a fruitful level of abstraction/concreteness that would further its interdisciplinary analyses and understanding. What is lacking is an approach that will bring algorithm studies together with other disciplines where discussions of fairness have a long history, such as law. In the context of administering justice, fairness has earlier been productively employed by access to justice studies, in order to consider both structural and contextual challenges as well as their intersectionality. Hence, we suggest such a more holistic and interdisciplinary approach to algorithmic fairness (combining multiple domain empirical and theoretical study) could contribute to a better understanding of fairness and ways to further it. Algorithmic fairness offers one area in which to show how – at the end of the day – the issues of any new technology are still largely related to broader discussions of values (Luusua and Ylipulli, 2020; Koulu, 2021).

## 4. Algorithmisation in the context of technological and legal social ordering

In the section above we considered some problems and solutions recently discussed in relation to the algorithmisation of society. We collected points that relate to how results of algorithmic systems could be potentially contested and how they could be overseen in order to maintain or improve fairness in administering justice. Moreover, we suggested that studying algorithmic fairness could advance by modeling itself after more classic ways of examining access to justice, in both style and method. However, the fields of algorithmisation and access to justice studies relate also in substantive terms, as much of the logic behind introducing algorithmic systems into administering justice aims at improving access to justice.

What, then, are the connections between law and algorithmisation and how does technological change influence law and *vice versa*? Society's algorithmisation is defined as a distinct form of social ordering connected with reliance on autonomous data-driven computational predictions (Aneesh, 2009; Gillespie, 2014). Much of legal research discusses algorithmisation in terms of algorithmic or automated decision making (ADM), terminology also adopted in the EU's General Data Protection Regulation (679/2016, GDPR) (Buiten, 2019, p. 10; Castets-Renard, 2019; Finck, 2020). Understandably, many legal scholars have been interested in automation of legal decision making, i.e., the decision-making processes in the courts or other public bodies. Another strand has focused on issues related to regulating algorithm use in society (Kaminski, 2019; e.g. Brkan and Bonnet, 2020). Both of these aspects ultimately deal with what should be considered fair and just, and how such fairness is produced and administered. Even if studied in the legal decision-making context, legal perspectives on fairness can also inform acceptable algorithm use beyond legal processes.

For legal processes, algorithmisation entails a promise of better and faster justice. On the one hand, both court proceedings and out-of-court conflict management systems are considered to gain from the inherent promises of algorithmisation, namely cost and time savings, and increased efficiency and accessibility provided by automation and online systems. Algorithmic tools and digital technologies are considered to entail possibilities to improve access to justice on the level of individual conflicts by the online dispute resolution (ODR) community. Additionally, they are considered to legitimize systems that deal with justice – not only courts, but administrative institutions as well – by creating transparency and trust in the processes (see e.g., Abdel Wahab et al., 2012). For example, the promise of speedy resolution is particularly enticing for judicial systems bogged down by a backlog of cases of small financial interest (see e.g., Zeleznikow, 2017; Sourdin, 2018; Schmidt-Kessen et al., 2020). However, on the other hand, law also defines the limits of acceptable algorithm use by pre-existing criteria set by the legal principles of due process and good administration. For example, in Finland at the end of 2019, the Parliament Deputy Ombudsperson found that Tax Administration's ADM use was unconstitutional and did not fulfill the requirements of good governance (e.g., informing taxpayers they were subject to ADM) (Parliamentary Ombudsman of Finland, 2019).

Algorithmisation is often considered to create new problems in need of new solutions. At the moment, more or less piecemeal new regulatory proposals are being drafted as a reaction to algorithmisation. However, the different sectors of society affected by algorithmisation are already regulated. Hence, national and transnational legal orders are defining the limits of acceptable and unacceptable use of algorithmic systems across different sectors based on existing law. Courts produce case law interpreting existing laws, and legal scholars develop the black letter doctrine of data protection and AI liability as well as conceptualisations of interplay between law, technology, and society (Cohen, 2019).

Simply put, law is a focal societal mechanism that defines, enables, and constrains algorithmisation. Hence, it matters how these issues are framed within different legal practices. For as long as algorithmisation is not framed as and dealt with as a more systemic issue, the solutions offered for the issues it raises are patchy at best. For example, the current ubiquity of data protection and privacy concerns related to digital technologies can at least partly be attributed to the implementation of GDPR in May 2018. Such a fragmented development of the legal social ordering risks furthering sectoral siloes within law as well as society. The demand for fair, lawful, and ethical use of algorithms has led to acknowledging the need for interdisciplinary research to address associated challenges (e.g., Pasquale, 2015; O'Neil, 2016; Hagendorff, 2020). Although the need for such dialogue and debate is broadly acknowledged, research often remains fragmented into disciplinary siloes (Yeung and Lodge, 2019). The method for overcoming this remains under-researched.

Algorithmisation does not simply change legal processes or conflict management it also changes conflicts themselves: the conflicts increasingly emerge within digital structures and online environments. This requires adjustments to conflict management mechanisms that were traditionally built around people being physically present (on dispute resolution and technological change in general, see e.g. Koulu, 2019). For example, traditional redress mechanisms are not effortlessly suited to provide legal protection in novel types of conflicts, such as algorithmic discrimination. Furthermore, it is difficult if not impossible to translate fairness and justice, as they are defined by law, into algorithmic systems (see e.g., Koivisto, 2020; Koulu, 2020b; Hakkarainen, 2021; Wachter et al., 2021). Moreover, the growing reliance on technology can also amplify the digital divide: for people with no access or knowledge to navigate the digital environment it becomes harder than before to partake in processes leading to important decisions concerning them (see e.g., Rabinovich-Einy and Katsh, 2017; Wing, 2018; Toohey et al., 2019).

However, these dynamics of change and the inadequacies of legal redress are not completely unprecedented. While technology changes some aspects of conflicts, others remain the same: the diachronic continuum and dynamic nature of technological change is often ignored in current legal research. It also matters greatly whether technological change is studied in the abstract or empirically, and whether it is studied from the point of view of those wielding societal power or from the perspective of the individuals subjected to that power. We frame algorithmisation as part of the broader context of the computational turn, a term used in digital humanities to describe the scientific shift toward the adoption of methods borrowed from computer science (on the philosophical understanding of the computational turn, see Hildebrandt and de Vries, 2013). However, we use the term in a more socio-legal meaning to refer to the growing reliance on computer-based technologies, their underlying logic, and associated rationalities and practices since the 1940's. This framing also remedies the false assumptions about the apolitical and ahistorical characteristics often associated with technological developments aligning with perspectives of Science and Technology Studies, STS (Winner, 1985; Jasanoff, 2004; Feenberg, 2017; Koulu, 2021).

In sum, when algorithmisation is studied in the more systemic frame of technological and legal social ordering, it becomes clear that structural problems of inequality are not novel or customary only to algorithmic decisions. This, together with the understanding that technological change is constant and the choice of perspective matters greatly, all spring to mind access to justice studies. Hence, our premise is the observation that similar issues and problematisations as those examined within algorithm studies have long been researched and debated under the general title of access to justice. That is, similar discussions have now resurfaced in a different context of societal change, begging the question as to what extent the old challenges are amplified by algorithmisation. To us it seems clear that algorithm studies, including AI ethics discussions, actually relate to and concern fairness and justice. These are also evergreen topics of access to justice research that have untapped potential within the algorithmic context. Despite differences in time and context, there are many similarities between the discussions on access to justice and algorithmisation. This we hope to demonstrate in the next section (3) through our examples selected from algorithm studies.

It is highly probable that prior research on these issues – fairness and justice examined from the access to justice perspective within law and beyond it – can offer useful insights for algorithm studies, especially if undertaken interdisciplinarily. Therefore, in the last section we introduce an idea of a methodological approach such studies could apply.

## 5. Suggested methodological approach to study algorithmic fairness interdisciplinarily

As we suggested at the outset, the dynamics of change and the inadequacies of legal redress that arise out of algorithmisation are not completely unprecedented. To draw on the diachronic continuum and the dynamic nature of technological change often ignored in current (legal) research, we suggest looking back – also to past research to find best practices. Looking back, we identified access to justice studies as worth examining for its scientific and societal impact. But like our examples of transparency and human oversight depict, research using the same terminology is not enough to address the social and legal implications of algorithmisation. In addition, there is need for methodological reflection, tools of which we discuss briefly in this section.

To research (map, understand, and theorize) the ongoing transformation in the algorithmisation of justice, many domains need to collaborate. To name a few such domains, law, philosophy, social sciences generally (specifically sociology), and computer science (especially Human-Computer Interaction studies) need to cooperate. But what does it mean in practice to collaborate interdisciplinarily in order to reap the benefits of combining algorithmic fairness with access to justice? To succeed, that is to go beyond multidisciplinary research, interdisciplinary research requires some sort of shared approach for participating domains. In our own interdisciplinary research endeavors, we have recognized several challenges. Starting collaboration from defining a common concept, model or theory, constructing shared vocabulary, and negotiating a joint understanding of the research problem prior to undertaking any actual research is unmotivating, time-consuming and in cost-benefit terms an inefficient way of researching a given issue. Often a shared starting point, an encompassing yet useful common concept or theory to base deductive interdisciplinary research on, is and remains lacking. Hence interdisciplinary research endeavors seem persistently divided by theory and methodology instead of being unified by them. Nevertheless, interdisciplinary collaboration is generally deemed especially pertinent for studying new phenomena that can be described as complex or even a wicked (societal) problem – such as algorithmisation.

How, then, would we suggest organizing interdisciplinary research on algorithmic fairness? We suggest bringing together different disciplines to collaborate with one another, to learn and gain new knowledge from each other. The idea is to share the interdisciplinary venture of doing research, hopefully but not necessarily contributing common deliverables together. More importantly, the approach we envision facilitates mobilizing knowledge while *doing* researching instead of afterwards, from which each discipline gains its own added value. We envision an approach that enables coming together across disciplinary borders to study a given phenomenon, compare, share and reflect on data, analytical frameworks or methods applied to gain theoretical and empirical knowledge. However, such collaboration also allows each discipline to hold on to its own epistemic conventions. Hence we would expect multi-domain research collaboration to aim for interdisciplinarity, understood as something beyond the fallback option of multidisciplinary but short of transdisciplinary research.

To bypass the need for other shared starting points than the phenomenon studied, we suggest a combination of grounded theory (GT) and boundary object as common research method. The empirical enquiry and theory development that brings together several disciplines can proceed differently from more mainstream concept-/theory-based deduction. One option is to combine using the (workplace ecology and computer science inspired) sociological methodological concept of boundary object (Star, 1988; Star and Griesemer, 1989) with Grounded Theory (Glaser and Strauss, 1967; Charmaz, 2006).

Grounded theory (GT) is a qualitative empirical approach developed in social sciences, in which theory (or model) is developed out of the collected materials and not the other way round. Most research is organized hypothetico-deductively, rather than inductively like GT. As an inductive approach, GT does not start with a hypothesis to verify or falsify, or a theory to apply and assess. GT is called ‘grounded’ as theory is developed from the reality it aims to explain (Charmaz, 2006). Free from straightjackets of existing theories or preconceptions, GT fits the study of emerging subjects, new situations and activities in turbulent circumstances, and introduces novel perspectives on existing subjects and activities. Hence, GT could be useful way to approach algorithmic fairness and develop its theoretical and practical connections with access to justice.

What is then the starting point for GT approach to algorithmic fairness? We propose using access to justice as a boundary object that brings together multi-domain research on algorithmic fairness. Paying attention to access to justice as a concept, or a value and goal, concretely requires paying heed to factors (e.g., technological, institutional, structural, economic) and constraints (e.g., physical, trust, time, cost) that hinder fair and equal access to justice. In the seminal work by Susan L. Star and James R. Griesemer, a boundary object is an object that resides between social worlds or groups which collaborate around the object without having a consensus on it (e.g., without agreeing on the meaning of a concept). Boundary objects enable interdisciplinary dialogue, as “they have different meanings in different social worlds but their structure is common enough to more than one world to make them recognizable, as a means of translation” (Star and Griesemer, 1989, p. 393). Hallmarks of a boundary object are interpretive flexibility and a specified scope of usage by a group (Star, 2010), going beyond what is often termed the natural language fuzziness of words (see e.g., Paunio and Lindroos-Hovinheimo, 2010). Later research on the use of boundary object suggests the notion has been used - though possibly not always in accordance with its original idea - to describe any interface mechanism between knowledge or actors (Trompette and Vinck, 2009, p. 16). For example, the notion of resilience has been considered boundary object in sustainability science (Brand and Jax, 2007). Boundary objects can take many forms, they can be abstract or concrete: we suggest under the abstract and general boundary object concept of access to justice, there is room for sharing more specific and concrete boundary objects, such as information (including concrete empirical datasets) across disciplines. Regardless of different social worlds missing consensus on the meaning of a concept, a boundary object can facilitate collaboration between several disciplines.

From our perspective, it is vital to include insights to legal research from other fields working on algorithmisation. This need is also further accentuated by the lack of empirical research. Legal studies' mainstream normative approach to law is doctrinal and theoretical rather than empirical. Yet even the fringe tradition of legal empirical research (Šadl and Holtermann, 2020) alone cannot provide an overview of the complex interaction of ways in which algorithmisation affects fairness or, as we put it more concretely, striving for fairness in access to algorithmic justice. At the same time, legal research can contribute additional understanding to other fields. This is because studying legal phenomena can be divided into an internal-to-law and external-to-law approach to them. As Kaarlo Tuori puts it: “legal scholar approaches the law from a participant's internal point of view, whereas the social scientist adopts an observer's external point of view.” (Tuori, 2002, p. 285) While the external-to-law approach may apply the same methodological tools as the rest of social or other sciences, when it is applied by a legal scholar it retains a special understanding of law as a symbolic and normative system.

Hence, there is untapped potential in studying algorithmisation, with law in the mix, to further understanding and to guide future technology governance and regulation. First, collaborating interdisciplinarily by building on boundary objects and grounded theory should manage to set aside the incessant as well as often consuming and unrewarding translating back and forth between scientific disciplines before, during, and after collaboration. Second, the potential to gain novel insight into phenomena is greater than when research is hypothetico-deductive or based on pre-existing theories or models.

## 6. Concluding remarks

In the above, we aimed for a two-fold contribution: first, furthering critical research on algorithmic fairness in the context of administering justice and, second, suggesting a methodological approach for interdisciplinary study of it. Our contribution was founded on the idea that in the context of administering justice, studies on algorithmisation in administering justice relate to and concern fairness. We connected the discussion on algorithmic fairness with that on access to justice. We offered concrete examples of algorithmic challenges and solutions based on which we argue that algorithm studies could learn a great deal from access to justice studies when debating transparency, explainability, accountability or human agency.

First of all, the perspective on algorithmic fairness could be changed to a more systemic one, as systemic problems require systemic solutions. Here the research field could learn from the approach of access to justice studies, which regularly studies phenomena both from an individual as well as from an institutional point of view. Second, studies on algorithmisation could methodologically draw on the varied interdisciplinary and methodological (empirical) points of view that have contributed to the success of access to justice studies in transforming law, science, and society. As one practical example context, we concluded that the role of human agency (HCI and interpersonal) is a more multifaceted feature of administering justice than studies seem to presume. Hence, it warrants further interdisciplinary research collaboration and empirical research on the deeper implications that practical changes to the role of human operators interacting with algorithmic systems have for access to justice of an individual.

Moreover, we contextualized algorithmisation in administering justice as the latest turn in centuries of technological evolution embedded in the computational turn that has affected societal structures. Hence, we also examined access to justice studies as a successful and impactful benchmark to study the effects of technological development in a historical context. We concluded that the systemic and historic context of research remains underdeveloped in studying algorithmic fairness.

Finally, we elaborated on a methodological approach that could facilitate such an exchange. One option is to employ access to justice as a boundary object to connect disciplinary perspectives and allow for interdisciplinary research collaboration while respecting disciplines' epistemic diversity. As a method to engage across disciplines we suggested using boundary objects possibly together with grounded theory (GT).

How is the perspective we suggest different from other law, technology, and society approaches? We hope to have shown that current algorithm studies remain rather ahistorical, apolitical, as well as sub-optimally siloed and fragmented. With a methodological approach that is fairly novel to many disciplinary domains, we have suggested alternative ways to do interdisciplinary research collaboration and hope to have shown feasible ways forward from the siloed disciplinary approaches to algorithmic fairness.

Our concern is that problems related to algorithmisation in administering justice are momentarily studied in isolation from each other. Hence the solutions suggested based on these studies are also piecemeal and fragmented at a practical and regulatory level, instead of addressing structural problems. However, many issues are not novel to new technology but go to the heart of democratic societies and legal systems. While quick fixes and one-size-fits-all solutions like adjusting already existing principles of non-discrimination and accountability can to an extent work – and some might say this is enough – the result can also be one of patchwork, leaving much to be desired if viewed through the lens of a more systemic approach to institutional and individual algorithmic fairness.

Hence, our conclusion is a call to arms under which we hope disciplines unite. We hope to have made it plain that further interdisciplinary research into algorithmic fairness is warranted. Algorithm studies have discussed several challenges and tentative solutions for increasing algorithmic fairness. However, we suggest that further interdisciplinary research on algorithmic fairness in administering justice could in many ways be modeled after access to justice studies. It should bring together philosophical, computer science, and administrative but also legal science to collaborate interdisciplinarily. Such a more holistic research approach has potential to make a significant contribution to algorithmic fairness.

## Author contributions

LK, RK, and SS contributed to the design and implementation of the research, to the analysis of the results, and to the writing of the manuscript. All authors equally contributed to the article and approved the submitted version.
